# Effects of Toki Shakuyaku San on Olfactory Function in a Postmenopausal Mouse Model

**DOI:** 10.7759/cureus.93444

**Published:** 2025-09-28

**Authors:** Masami Kumai, Mitsuharu Aga, Tomoko Ishikura, Takako Kanitani, Riho Minato-Yoshida, Takaki Miwa, Hideaki Shiga

**Affiliations:** 1 Otorhinolaryngology, Kanazawa Medical University, Ishikawa, JPN; 2 Otolaryngology, Kumai Clinic, Asahikawa, JPN; 3 Internal Medicine, Toyama Jonan Hospital, Toyama, JPN

**Keywords:** odor aversion behavior, olfaction, olfactory marker protein, ovariectomized mice, toki shakuyaku san

## Abstract

Objective: This study aimed to evaluate the effects of Toki Shakuyaku San (TSS) on olfactory function in a mouse model of sensorineural olfactory dysfunction, with a focus on the estrogen-deficient state. We investigated whether TSS promotes olfactory neuronal regeneration and recovery of odor-evoked behavior in both sham-operated and ovariectomized (OVX) mice to clarify its potential therapeutic value in postmenopausal olfactory impairment.

Methods: Female BALB/c mice underwent ovariectomy or sham surgery, followed by methimazole administration to induce olfactory epithelial injury. Mice were fed either a control diet or a diet containing 0.5% TSS. Odor aversion behavior was evaluated using a light-dark box, and olfactory marker protein (OMP) expression in the nasal epithelium was analyzed by immunohistochemistry on Day 14. Statistical analysis was performed using the Mann-Whitney U test.

Results: In sham-operated mice, those fed a TSS-containing diet exhibited significantly longer odor aversion time at day 14 when compared to the mice fed a control diet (P = 0.007). A significantly higher number of OMP-positive cells was observed in the superior nasal septum (R1) of TSS-fed mice than in control-fed mice (P = 0.03). In OVX mice, no significant differences were observed between the TSS-fed and control-fed groups in odor aversion behavior (P = 0.11) or in the number of OMP-positive cells in any of the four regions of the olfactory epithelium (P > 0.05).

Conclusion: Our results suggest that the effects of TSS on olfactory recovery may depend on the presence of estrogen, and its efficacy could be influenced by the hormonal status.

## Introduction

Olfaction is important for vital activities, such as food exploration, danger detection, memory recall, and emotional expression, in many organisms, including humans, and its dysfunction can reduce the quality of life and even lead to life-threatening situations [1]. For the treatment of sensorineural olfactory dysfunction, various pharmacological treatments, including zinc formulations, α-lipoic acid, vitamin A, minocycline, and theophylline, have been utilized in clinical practice [2]. There is currently no clear evidence-based standard treatment.

In Japan, Toki Shakuyaku San (TSS), an herbal medicine used for menopause, is conventionally used for treating sensorineural olfactory dysfunction [2]. We previously reported that the components of TSS, the *Atractylodes lancea* rhizome and Japanese angelica root, promote the secretion of nerve growth factor (NGF), a neurotrophic factor necessary for olfactory epithelial regeneration [3-7], from primary cultured astrocytes via the MAP kinase cascade [8]. Furthermore, an in vivo study using an olfactory dysfunction mouse model showed that NGF expression and olfactory epithelial regeneration were both enhanced in mixed feed containing TSS [8]. The induction of neurotrophic growth factor has also been found to be induced by the estrogen-like action of TSS [9, 10].

Menopause is a potential risk factor for sensorineural olfactory dysfunction, which is more prevalent in women aged approximately 55 years [11-13], with a higher incidence observed in postmenopausal women (6.2%) than in premenopausal women (3.5%). A previous study using a female ovariectomized (OVX) mouse model demonstrated that estrogen depletion induces suppressive effects on the regeneration of olfactory neurons [14]. Furthermore, estrogen may contribute to maintaining olfactory function by regulating neurotrophic growth factors [10]. Given that TSS exhibits estrogen-like effects [9] and enhances NGF expression in the olfactory system [8], the therapeutic effects of TSS on olfactory dysfunction might differ in postmenopausal women compared to those in other populations. However, its precise mechanism of action and efficacy in an estrogen-deficient state remain unclear.

In this study, we aimed to investigate the effects of TSS on olfactory function in a postmenopausal mouse model by evaluating olfactory neuronal regeneration and behavioral responses to odor stimuli.

## Materials and methods

Animals

Female BALB/c mice, seven weeks old, obtained from Sankyo Labo Service Corporation (Tokyo, Japan), were housed with a 12-hour light/dark rhythm and maintained at a constant temperature of 22-26°C. The mice were provided with standard solid food (CE-2; Crea Japan Inc., Tokyo, Japan) and water ad libitum. At the beginning of the experiment, the average weight was approximately 20-22 g in the mice.

Ovariectomy or sham operation

**Figure 1:**
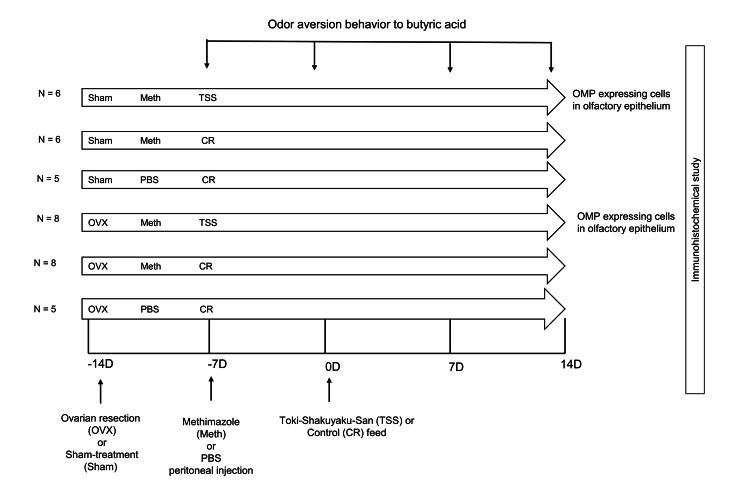
Experimental protocol Odor aversion behavior to butyric acid was assessed in 12 ovariectomized (OVX) mice and 12 sham-operated mice seven days after surgery. Following the assessment, mice received an intraperitoneal injection of methimazole and were fed either a 0.5% Toki Shakuyaku San (TSS) mixed diet or a control (CR) diet. Odor aversion behavior was assessed again on the day of diet initiation (Day 0) and on Days 7 and 14. On Day 14, mice were perfused with physiological saline and fixed with 4% paraformaldehyde under anesthesia. Additionally, five OVX mice and five sham-operated mice received phosphate-buffered saline (PBS) instead of methimazole. These mice were fed the CR diet starting on Day 0, and odor aversion behavior was assessed every seven days. OMP: olfactory marker protein

Seven-week-old female BALB/c mice underwent bilateral ovariectomy or sham operation under anesthesia with intraperitoneal administration of a mixed solution of medetomidine (0.75 μg/g), midazolam (4 μg/g), and butorphanol (5 μg/g) in phosphate-buffered saline (PBS) (Figure 1). For ovariectomy, both ovaries were identified within the fat pad through dorsal incisions and surgically removed. In sham-operated mice, the same dorsal incisions were made, and the ovaries were visually confirmed within the fat pad but not removed. The incision sites were then closed with sutures. The mice were awakened via intraperitoneal atipamezole administration 30 minutes after treatment.

Mixed feed incorporated with TSS or control feed was given seven days after methimazole administration.

Methimazole (75 mg/kg), which has been demonstrated to have an olfactory epithelial-damaging effect, was administered intraperitoneally to the eight-week-old mice (Figure 1). PBS was administered intraperitoneally to the ovariectomized (n = 5) and sham-treated mice (n = 5) (Figure 1). TSS was supplied by Tsumura & Co. (Tokyo, Japan) as a lyophilized powder. The pharmaceutical quality of TSS is controlled based on the 17^th^ edition of the Japanese Pharmacopoeia, which regulates the concentrations of (E)-ferulic acid, paeoniflorin, and atractylodiol [8]. TSS (0.5% w/w) was included in the animal feed (CE-2; Crea Japan, Inc.). The mixed feed containing TSS (0.5%) or the control feed was started seven days after administering methimazole. A control feed was provided for the PBS-administered mice.

Innate aversive response to butyric acid in mice

Odor aversion to butyric acid was evaluated by measuring the duration mice spent in the transparent compartment of a two‑chamber apparatus, as described elsewhere [15]. The test was recorded under infrared illumination with the apparatus covered by a dark plastic bag for the entire 3‑minute session. A cotton ball soaked in 300 µL of 10% butyric acid (in distilled water) was placed inside an Eppendorf tube attached to the closed compartment. Prior to testing, mice underwent two one‑minute habituation sessions in the apparatus without butyric acid.

We observed fluctuations in odor aversion behavior at −7, 0, seven, and 14 days relative to TSS administration (Day 0) (Figure 1). Odor-aversion behavior was assessed using a light-dark box. The box had a hole in the center that could be opened and closed; it could be moved between light and dark places. During the behavioral experiment, the box was covered to block vision from the outside, and a dark infrared camera was used to confirm the behavior in a dimly lit environment. A tube containing a cotton ball impregnated with a 10% butyric acid solution (a mouse repellent) was placed in a dark place, and the time spent in the light was counted. One-minute habituation periods were observed twice before the start of each day of the experiment. During the habituation period, the mice were allowed to move freely in a light/dark box, in which a tube containing a cotton ball soaked in distilled water was placed on the light/dark side.

Immunohistochemical detection of olfactory marker protein (OMP) in the olfactory epithelium

Immunohistochemical detection of OMP expression in the olfactory epithelium of sham-operated and ovariectomized mice was conducted on Day 14. Mice were anesthetized via intraperitoneal injection with a combination of medetomidine (0.75 µg/g), midazolam (4 µg/g), and butorphanol (5 µg/g), then perfused through the left ventricle with physiological saline, followed by fixation in 4% paraformaldehyde (FUJIFILM Wako, Osaka, Japan). Heads were dissected, the facial bones removed, and post-fixed overnight in 4% paraformaldehyde at 4°C. Nasal turbinates were excised, decalcified for seven days at 4°C in OSTEOSOFT® solution (MERCK KGAA, Darmstadt, Germany), and embedded in paraffin.

Paraffin sections of the nasal turbinates were deparaffinized in xylene, rehydrated through graded alcohols, and subjected to heat-mediated antigen retrieval in Tris‑EDTA buffer (pH 9.0) at 95 °C for 10-30 minutes. After blocking, sections were incubated with anti-OMP primary antibody (Fujifilm Wako, Osaka, Japan) at a dilution of 1:10,000 for one hour at 24°C. Following PBS washes, sections were treated with a biotin-conjugated secondary antibody for 30 minutes at 24°C before enzymatic detection using a peroxidase-based system (Histofine; Nichirei Biosciences, Tokyo, Japan). Normal goat serum served as the negative control in place of the primary antibody.

OMP-positive cells were manually counted along a 300 µm length of the basal membrane in each field. Two independent investigators (M.K. and T.K.) performed counts in four defined regions: R1, right superior nasal septum; R2, right medial turbinate; R3, right inferior nasal septum; and R4, right lateral turbinate. For each region, three coronal sections at 300 µm intervals, including the plane at the anterior edge of the olfactory bulb, were assessed. Mean values across the three sections per region were calculated and used for statistical analyses.

Ethics declaration

The study was performed in strict accordance with institutional and national guidelines for animal experimentation. All procedures were approved by the Animal Experiment Committee of Kanazawa Medical University, Ishikawa, Japan (approval numbers: 2020‑17 and 2023‑9). Ethical approval was required only for animal use; informed consent was not applicable.

Statistical analysis

Medians were compared using the Mann-Whitney U test. All statistical analyses were conducted with GraphPad Prism 8 (GraphPad Software, La Jolla, CA, USA). All reported P-values are two-tailed, and data are presented as medians with interquartile ranges (IQRs) unless otherwise specified. Statistical significance was defined as P < 0.05.

## Results

Recovery of odor aversion behavior using mixed feed containing TSS in sham-operated mice

**Figure 2:**
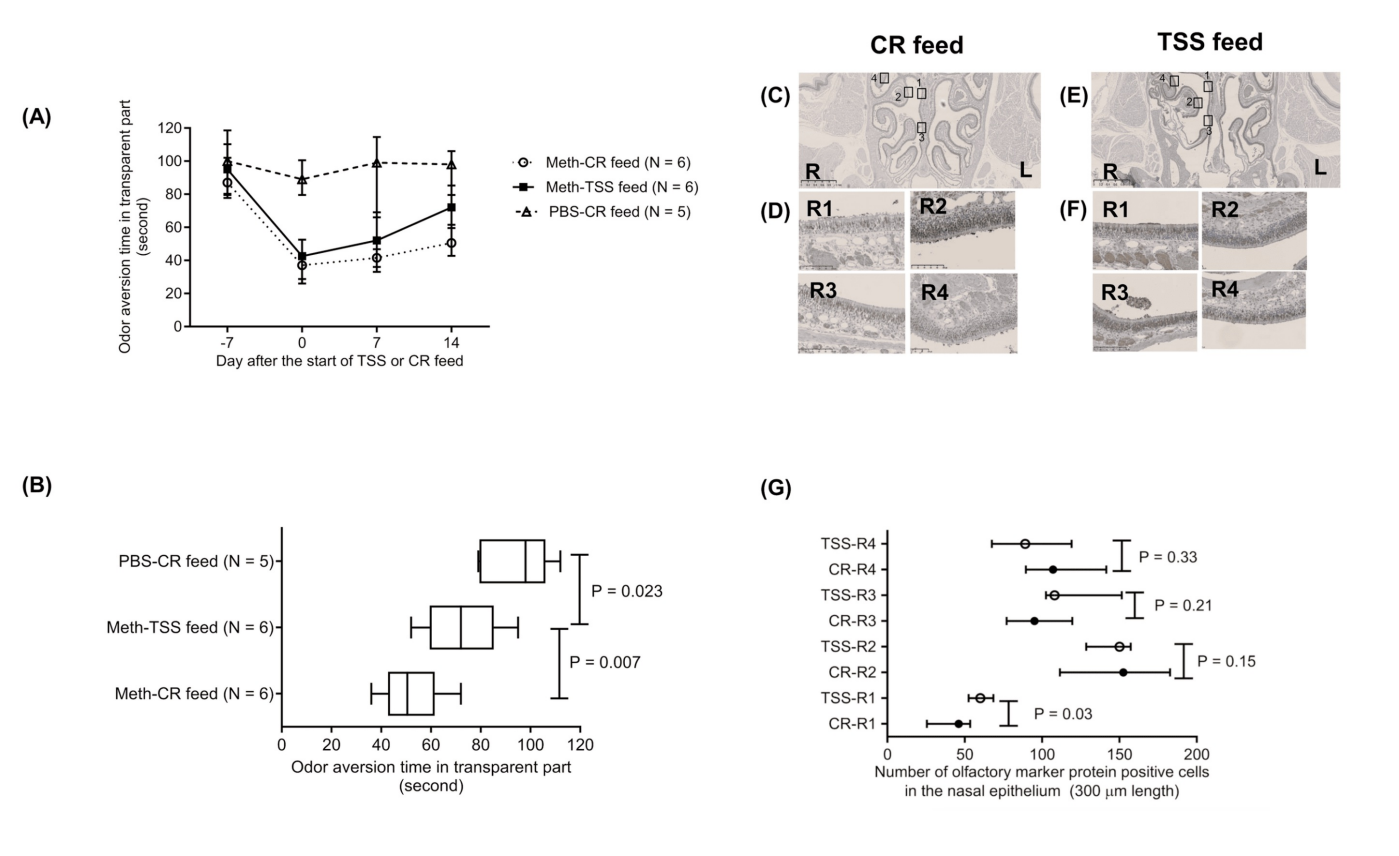
Odor aversion behavior and olfactory epithelium in sham-treated mice (A) Odor aversion time in the transparent compartment of the test box during a three-minute observation period in sham-operated mice fed a diet containing 0.5% Toki Shakuyaku San (TSS) or a control (CR) diet. Data are presented as medians with interquartile ranges. (B) Odor aversion time in the transparent compartment on Day 14 in methimazole (Meth)-injected sham-operated mice administered either the TSS or CR diet. A significant difference was observed between the mixed feed of TSS and CR feed in the sham-operated mice (P = 0.007). Furthermore, the odor aversion time on Day 14was significantly shorter in the methimazole-injected mice treated with the mixed feed of TSS than the phosphate-buffered saline (PBS)-injected mice treated with CR feed (P = 0.023). Significance was determined using the Mann–Whitney U test. (C–F) Representative coronal sections of the right nasal cavity obtained from sham-operated mice on Day 14:(C, D) CR-fed mice; (E, F) TSS-fed mice. Olfactory marker protein (OMP)-positive cells were visualized by immunohistochemical staining. The right-side olfactory epithelium was divided into four regions: R1, the superior part of the nasal septum; R2, the medial turbinate; R3, the inferior part of the nasal septum; and R4, the lateral turbinate. Scale bars = 1 mm (C, E) and 100 μm (D, F). (G) Number of OMP-positive cells within a 300-μm segment of the right-side nasal epithelium in sham-operated mice on Day 14. In Part 1 (R1), the number of OMP-positive cells was significantly higher in the TSS-fed group compared to the control-fed group (P = 0.024). However, no significant differences were observed between the groups in Parts 2, 3, and 4.

In sham-operated mice, the duration spent in the transparent compartment, indicative of odor aversion, declined by Day 7 following intraperitoneal methimazole administration (Figure 2A). Conversely, PBS-treated controls receiving standard feed (n = 5) exhibited no attenuation of innate butyric acid aversion at any observed time point (Figure 2A). Fourteen days after initiation of TSS or control feeding, mice on the TSS diet (n = 6) demonstrated significantly stronger aversive responses to butyric acid compared to those receiving control feed (n = 6; P = 0.007; Figure 2B).

Immunohistochemical assessment of OMP in the nasal epithelium of sham-operated mice following 14 days of TSS or control diet

The number of OMP-expressing cells in the olfactory epithelium was assessed. The numbers were examined on Day 14 when the effects of the feed were confirmed. We divided the olfactory epithelium on the right side of the nasal epithelium into four parts in sham-treated mice and counted the number of OMP-expressing cells in the nasal epithelium (Figure 2C-2F). In part 1 (R1), an increase in the number of OMP-expressing cells was observed in the TSS mixed feed group compared with those in the control feed group (P=0.03; Figure 2G). However, no significant differences were observed between the groups in Parts 2, 3, and 4 (Figure 2G).

Recovery effect of odor aversion behavior using mixed feed incorporated with TSS in OVX mice

**Figure 3:**
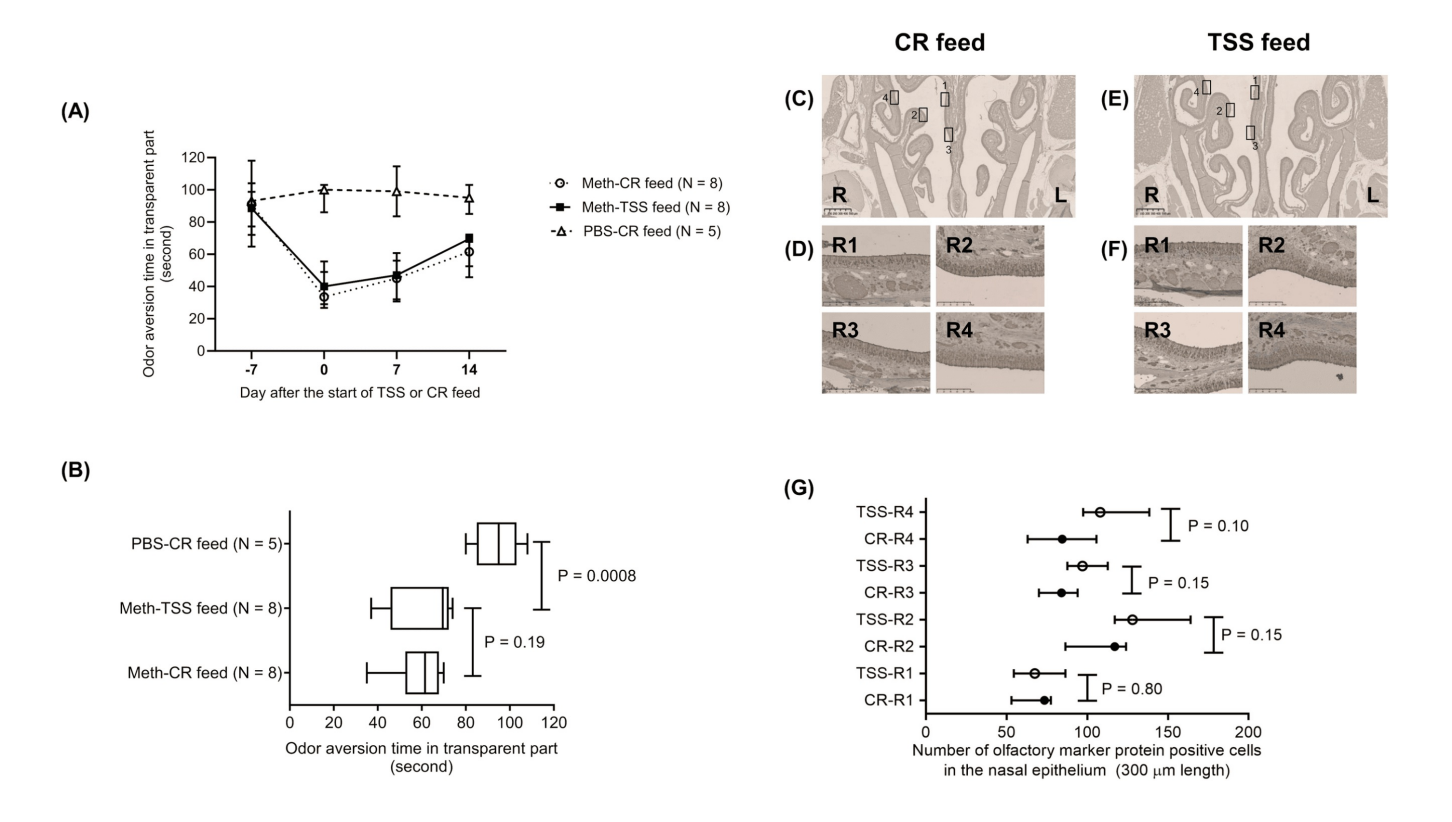
Odor aversion behavior and olfactory epithelium in OVX mice (A) Odor aversion time in the transparent compartment of the test box during a three-minute observation period in ovariectomized (OVX) mice fed a diet containing 0.5% Toki Shakuyaku San (TSS) or a control (CR) diet. Data are presented as medians with interquartile ranges. (B) Odor aversion time in the transparent compartment on Day 14 in methimazole (Meth)-injected OVX mice administered either the TSS or CR diet. No significant difference was observed between the mixed feed of TSS and CR feed in the OVX mice (P = 0.19). In contrast, the odor aversion time at 14 days was significantly shorter in the methimazole-injected mice treated with the mixed feed of TSS than the phosphate-buffered saline (PBS)-injected mice treated with CR feed (P = 0.0008).

In OVX mice, odor aversion, measured by time spent in the transparent chamber, declined on Day 7 following intraperitoneal administration of methimazole (Figure 3A). In contrast, PBS-treated controls fed a standard diet (n = 5) maintained consistent innate aversion to butyric acid at all time points (Figure 3A). By Day 14, there was no significant difference in butyric acid aversion between TSS-fed mice (n = 6) and control-fed mice (n = 6) (P = 0.11; Figure 3B). These observations suggest that ovariectomy impairs the efficacy of TSS in rescuing innate odor aversion following olfactory epithelial injury.

Immunohistochemical assessment of OMP in the nasal epithelium of OVX mice following 14 days of TSS or control diet

We assessed the number of OMP-expressing cells in the olfactory epithelium of OVX mice 14 days after the start of TSS or control feed (Figures 3C-3F). The number of OMP-expressing cells in Parts 1, 2, 3, and 4 of the OVX mice revealed no significant difference between the TSS mixed feed and control feed groups (Part 1: P=0.80; Part 2: P=0.15; Part 3: P=0.15; Part 4: P=0.10; Figure 3G). These findings indicate that the effects of TSS on the recovery of OMP-expressing cells in the olfactory epithelium after damage decreased after ovariectomy in mice.

OVX-sham comparisons on Day 14

On Day 14, differences between sham and OVX under CR and TSS were evaluated in behavioral assay and immunohistochemistry. In the behavioral assay, we did not detect a significant difference, although within the TSS condition, the sham group tended to show longer avoidance than the OVX group (median (IQR): Sham 43.1 (42.2-45.1) vs. OVX 39.7 (37.2-41.0); P=0.13). For OMP counts, we observed no significant differences in any of the four regions. Detailed results are provided in Appendix A.

## Discussion

In this study, we found that including TSS in the diet markedly enhanced the restoration of odor aversion behavior and the expression of OMP in the olfactory epithelium following methimazole-induced damage in sham‑operated mice. Notably, these beneficial effects of TSS were attenuated in ovariectomized mice.

We have demonstrated that TSS promoted the recovery of olfactory function following methimazole-induced damage, as evidenced by improved odor aversion behavior and increased expression of OMP in the olfactory epithelium. This finding is consistent with that of previous clinical studies demonstrating the efficacy of TSS in patients with post-infectious olfactory dysfunction [16]. To explore the underlying mechanism, our in vitro findings revealed that TSS and its major herbal components, *Atractylodes lancea* rhizome (ALR) and Japanese angelica root, significantly enhanced the production of NGF, a member of the neurotrophin family that supports neural survival after brain damage [17], in cultured astrocytes [8]. Previous reports have identified active compounds, such as atractylodin and levistolide A, in these herbs, which contribute to NGF induction [8]. Although the precise mechanisms remain to be fully elucidated, the current evidence suggests that TSS may facilitate olfactory recovery by activating NGF-related regenerative pathways.

However, in OVX mice, these effects of TSS were not clearly detected, indicating that estrogen depletion attenuates responsiveness to TSS. This result might be explained by the importance of estrogen in the regeneration of olfactory neurons. A previous study revealed that ovariectomy suppressed the expression of OMP, Ki-67, and TrkA in the olfactory epithelium during the recovery phase [14]. Although the NGF level increased in the olfactory bulb, sufficient regeneration was not promoted in estrogen-depleted mice. Estrogen presumably helps not only NGF production but also its function in the regeneration process. In support of this, a previous report demonstrated that estrogen and neurotrophins such as NGF can act on the same neurons in the basal forebrain and may work synergistically to regulate neuronal survival, differentiation, and regeneration [18]. In our results, TSS did not show enough effect in OVX mice; thus, estrogen may be required for the effective function of TSS in damaged olfactory tissue.

Interpretation of OVX-related group differences hinges on assessment timing, dosing schedule, and cohort timing. At Day 14 after TSS administration, no significant differences were detected between sham and OVX in either the TSS or control cohorts. Our TSS dosing schedule also differed from prior work [8]. They initiated TSS on Day 3 after methimazole and observed an OMP difference on Day 14 after methimazole, whereas we initiated TSS on Day 7 after methimazole, to align with the onset of olfactory epithelial regeneration, and compared OMP on Day 21 after methimazole to focus on TSS-related regenerative effects. Additionally, the OVX and sham cohorts were conducted in different calendar windows, so minor differences in growth or environmental conditions cannot be fully excluded.

Among herbal medicines used for olfactory dysfunction, TSS is currently the only formulation for which a possible mechanism, enhancement of NGF-mediated neuronal regeneration, has been experimentally suggested. Other herbal medicines, such as Ninjinyoeito and Kamikihito, have also been used clinically in Japan. However, their mechanisms of action remain unclear [19]. In cases where TSS is ineffective, alternative approaches, such as the use of other herbal medicines or olfactory training, may be considered. Nevertheless, olfactory training is likely to be less effective in the early stages of recovery, when the number of mature olfactory neurons is still limited. In particular, postmenopausal olfactory dysfunction may be more refractory due to reduced neuroregenerative capacity. These considerations demonstrate the importance of conducting further studies to develop effective therapeutic strategies, especially for estrogen-deficient populations.

The limitations of this study are that we did not assess the effects of TSS on olfactory epithelial regeneration in ovariectomized mice under estrogen replacement conditions, because animal experimental estrogen handling is considered hazardous in our animal testing facilities. We used the OVX model to mimic estrogen deficiency, but did not directly measure estradiol levels or uterine atrophy in all animals. Thus, our interpretations assume that the OVX procedure effectively induced estrogen deficiency. Future studies should include these verifications to strengthen the conclusions.

One potential limitation of the behavioral assay used in this study, the odor aversion test in the light-dark box, is that it may not solely reflect olfactory function. The results could also be influenced by factors such as anxiety or general activity levels, which may affect the animal's behavior independently of its olfactory ability. Although this test is commonly used to assess olfactory dysfunction, we acknowledge that the observed effects may be influenced by non-olfactory factors. Future studies could employ additional assays or controls, such as measuring baseline activity or anxiety levels, to more specifically isolate the contribution of olfactory function to the observed behavior.

Only the right-sided nasal epithelium of mice was assessed for OMP expression, because the mice were fed orally. Importantly, OVX alone did not completely suppress potential TSS effects. Further studies are warranted to clarify the detailed molecular pathways and explore the potential of TSS as a treatment option for sensorineural olfactory disorders. We did not perform zone marker staining or odorant receptor mapping, so our assignment of part 1 to zone 1 is tentative. However, anatomically, part 1 is located dorsally/medially, consistent with classical zone 1 (NQO1/OMACS positive, OCAM negative). Because the change in OMP‑positive cell number was observed only in part 1, it is plausible that olfactory sensory neurons (OSNs) in the zone 1 region are selectively vulnerable to our experimental treatment. Zone 1 has been reported in prior studies to be more sensitive to metabolic or oxidative stress [20], which may explain the regional specificity. Given this, one may hypothesize that some butyric acid-responsive odorant receptors are enriched in zone 1. If so, impairment in zone 1 OSNs could disproportionately affect butyric acid detection. This remains speculative, and future work should include co‑staining with NQO1/OMACS/OCAM and odorant receptor expression mapping, as well as functional assays comparing dorsal versus ventral OSN responsiveness.

## Conclusions

Our findings suggest that TSS promotes the regeneration of olfactory neurons and functional recovery after chemical-induced damage, at least partly through the enhancement of NGF signaling. This study provides behavioral evidence, improved odor-aversion performance under controlled conditions, demonstrating TSS efficacy, which represents a novel contribution beyond histological endpoints. The diminished efficacy of TSS in OVX mice implies that estrogen may play a synergistic role in this regenerative process. These results not only support the clinical utility of TSS in sensorineural olfactory dysfunction but also highlight the need to consider the hormonal status when evaluating therapeutic responses.

## Appendices

Appendix A 

**Table 1:** Comparisons on Day 14 between sham-operated and ovariectomized (OVX) groups treated with Toki Shakuyaku San (TSS) or control (CR) for odor-aversion behavior and OMP-positive cell counts were performed using the two-sided Mann–Whitney U test.

Measure	Location	Condition	Sham (n=6)	OVX (n=8)	U	p
Behavior, seconds, median (IQR)		CR	50.5 (46.3–56.3)	61.5 (57.5–67.3)	15.5	0.3
		TSS	72.0 (63.5–80.5)	69.5 (59.3–70.8)	31	0.4
OMP, cells/section, median (IQR)	R1	CR	46.0 (30.0, 53.0)	73.5 (62.0, 76.0)	3	0.06
		TSS	60.0 (58.0, 65.0)	67.5 (57.0, 71.5)	10	0.69
	R2	CR	152.5 (144.0, 178.0)	117.0 (99.0, 120.5)	21	0.1
		TSS	150.0 (131.0, 156.5)	128.0 (118.5, 159.0)	14	0.84
	R3	CR	95.0 (77.0, 109.0)	84.0 (74.5, 86.0)	18	0.29
		TSS	108.0 (107.0, 136.0)	97.0 (92.5, 102.5)	21	0.1
	R4	CR	107.0 (90.0, 120.0)	84.5 (75.0, 92.0)	20	0.15
		TSS	89.0 (74.0, 114.0)	108.0 (107.5, 121.5)	8	0.42
